# Inhibition of miR‐103a‐3p suppresses lipopolysaccharide‐induced sepsis and liver injury by regulating FBXW7 expression

**DOI:** 10.1002/cbin.11372

**Published:** 2020-05-12

**Authors:** Yu‐Ping Zhou, Qin Xia

**Affiliations:** ^1^ Department of Anesthesiology Shanghai Dermatology Hospital, Tongji University NO. 1278, Bao‐de Road Shanghai China; ^2^ Department of Anesthesiology Tenth People's Hospital, Tongji University NO. 301, Yan‐Chang‐Zhong Road Shanghai China

**Keywords:** FBXW7, liver failure, LPS, miR‐103a‐3p, sepsis

## Abstract

Inflammation, apoptosis, and oxidative stress are involved in septic liver dysfunction. Herein, the role of miR‐103a‐3p/FBXW7 axis in lipopolysaccharides (LPS)‐induced septic liver injury was investigated in mice. Hematoxylin‐eosin staining was used to evaluate LPS‐induced liver injury. Quantitative real‐time polymerase chain reaction was performed to determine the expression of microRNA (miR) and messenger RNA, and western blot analysis was conducted to examine the protein levels. Dual‐luciferase reporter assay was used to confirm the binding between miR‐103a‐3p and FBXW7. Both annexin V‐fluoresceine isothiocyanate/propidium iodide staining and caspase‐3 activity were employed to determine cell apoptosis. First, miR‐103a‐3p was upregulated in the septic serum of mice and patients with sepsis, and miR‐103a‐3p was elevated in the septic liver of LPS‐induced mice. Then, interfering miR‐103a‐3p significantly decreased apoptosis by suppressing Bax expression and upregulating Bcl‐2 levels in LPS‐induced AML12 and LO2 cells, and septic liver of mice. Furthermore, inhibition of miR‐103a‐3p repressed LPS‐induced inflammation by downregulating the expression of tumor necrosis factor, interleukin 1β, and interleukin 6 in vitro and in vivo. Meanwhile, interfering miR‐103a‐3p obviously attenuated LPS‐induced overactivation of oxidation via promoting expression of antioxidative enzymes, including catalase, superoxide dismutase, and glutathione in vitro and in vivo. Moreover, FBXW7 was a target of miR‐103a‐3p, and overexpression of FBXW7 significantly ameliorated LPS‐induced septic liver injury in mice. Finally, knockdown of FBXW7 markedly reversed anti‐miR‐103a‐3p‐mediated suppression of septic liver injury in mice. In conclusion, interfering miR‐103a‐3p or overexpression of FBXW7 improved LPS‐induced septic liver injury by suppressing apoptosis, inflammation, and oxidative reaction.

**Abbreviations**
AAV: adeno‐associated virus
CAT: catalase
DMEM/F‐12: Dulbecco's modified Eagle medium F‐12
FBXW7: F‐Box and WD repeat domain containing 7
FITC: fluoresceine isothiocyanate
GAPDH: glyceraldehyde‐3‐phosphate dehydrogenase
GSH: glutathione
H&E: hematoxylin‐eosin staining
IL‐1β: interleukin 1β
IL‐6: interleukin 6
LPS: lipopolysaccharides
mut: mutant
PI: propidium iodide
qRT‐PCR: quantitative reverse‐transcription polymerase chain reaction
ROS: reactive oxygen species
SOD: superoxide dismutase
TNF‐α: tumor necrosis factor

## 1 INTRODUCTION

Sepsis has been considered as a systemic inflammatory response syndrome and also a chief cause of organ failure, including liver injury (Hattori, Hattori, Suzuki, & Matsuda, 2017; Jiang et al., 2018; Mann, Baun, Meininger, & Wade, 2012). Lipopolysaccharide (LPS) is the main component of endotoxin, as it always causes inflammatory responses and oxidative stress, resulting in acute progression of liver dysfunction (Boe et al., 2010). LPS‐induced sepsis mice have been applied as an acute liver injury model, mainly in molecular mechanism researches (Z. Zhang et al., 2001). Septic liver failure contains complex pathophysiological changes, including reactive oxygen species (ROS), apoptosis, and inflammation (Bernal, 2017; Hattori et al., 2017). The oxidation‐caused liver damage is due to abnormality in antioxidative enzymes, such as catalase (CAT), glutathione (GSH), and superoxide dismutase (SOD; Andrades et al., 2011). Furthermore, LPS‐induced liver failure is often coupled with the release of inflammatory factors, such as tumor necrosis factor (TNF‐α), interleukin‐6 (IL‐6), and interleukin‐1β (IL‐1β; Dan et al., 2015; Zhong et al., 2016). Additionally, Bax and Bcl‐2 are essential indicators of cell apoptosis in various diseases including liver failure (Hattori et al., 2017; Jiang et al., 2018; Soares e Silva et al., 2015). Nevertheless, the precise molecular mechanism of sepsis is complex and unclear, and it needs to be further studied.

MicroRNAs (miRNAs) present single‐stranded noncoding RNAs, and they always target messenger RNA (mRNA) to inhibit translation (Giza et al., 2016; Roberts and Steer, 2010). A variety of miRNAs have been involved in sepsis‐induced organ failure including kidney failure, cardiac dysfunction, lung injury, and liver injury (Kingsley and Bhat, 2017; Wang et al., 2013, W. Zhang et al., 2019). For example, miR‐191‐5p attenuates sepsis‐induced kidney injury via binding to oxidative stress‐responsive 1 (Qin, Wang, & Peng, 2019), and miR‐106a enhances sepsis‐mediated acute kidney failure by targeting THBS2 (Shen, Yu, Jing, & Zhang, 2019). Next, miR‐146a inhibits sepsis‐induced myocardial dysfunction by targeting ErbB4 (An, Feng, Xi, Xu, & Sun, 2018), and miR‐21‐3p modulates sepsis‐induced cardiac dysfunction by targeting SORBS2 (Wang et al., 2016). Then, miR‐203 ameliorates septic lung injury via targeting VNN1 (Ling, Lu, Wang, Shen, & Zhang, 2019), and miR‐802 attenuates LPS‐induced acute lung injury by targeting Peli2 (You et al., 2019). Furthermore, miRNAs are also involved in sepsis‐associated liver injury. For instance, miR‐30a suppresses proliferation and increases apoptosis of hepatocytes by regulating SOCS‐1‐mediated JAK/STAT pathway in sepsis rats (Yuan et al., 2019), and MCPIP1‐regulated miR‐9/SIRT1 axis is involved in LPS‐induced liver injury (Han et al., 2019). These previous studies indicate that miRNAs have an essential role in sepsis‐associated organ injury (or failure), including liver failure. Consequently, investigating the roles of miRNAs in sepsis‐induced liver injury would offer a better understanding of the potential molecular mechanism in septic liver failure. In this study, we selected to study the role of miR‐103a‐3p in LPS‐induced septic liver by performing GEO2R bioinformatic analysis. The following experiments showed that miR‐103a‐3p was upregulated in the serum and liver of sepsis mice, and downregulation of miR‐103a‐3p could improve sepsis‐induced liver injury. In the existing studies, miR‐103a‐3p has always been shown to function as an oncogene in human gastric cancer (Hu et al., 2018), breast cancer (Xiong, Lei, Zhang, & Fu, 2017), and colon‐rectal carcinoma (Hong, Feng, Sai, & Tao, 2014). Additionally, miR‐103a‐3p is upregulated in the plasma of hypertension patients (Karolina et al., 2012) and it is also elevated in the urine of diabetes mellitus patients (Bacon, Engelbrecht, Schmid, Pfeiffer, & Gallagher, 2015), and moreover, miR‐103a‐3p promotes angiotensin II‐induced renal inflammation and fibrosis by modulating SNRK/NF‐κB/p65 signaling (Lu et al., 2019), suggesting that miR‐103a‐3p might also aggravate septic liver injury. However, whether and how miR‐103a‐3p controls liver dysfunction remains unknown.

To investigate the potential molecular mechanism of miR‐103a‐3p in septic liver injury, we predicted the downstream target genes of miR‐103a‐3p using *TargetScan tools*. In this study, we observed that FBXW7 was a direct target of miR‐103a‐3p. In the previous studies, FBXW7 often acted as a tumor suppressor in hepatocellular carcinoma (Imura et al., 2014), colorectal cancer (Fiore et al., 2019; Li et al., 2019), non‐small‐cell lung cancer (Xiao et al., 2018), breast and ovarian cancer cells (Zhao, Wang, Mu, Xu, & Sang, 2017), and others. Most importantly, FBXW7 suppresses inflammatory signaling via decreasing C/EBPδ and its target gene TLR4 (Balamurugan et al., 2013), and FBXW7 inhibits hepatic inflammation and insulin resistance by repressing HMGB1‐mediated innate immune pathway in nonalcoholic fatty liver disease (C. Zhang et al., 2019), implying that FBXW7 might act as a suppressor of septic liver injury. Consistently, our data demonstrated that overexpression of FBXW7 significantly attenuated LPS‐induced liver injury in mice. In the present study, we collected the serum from septic patient and LPS‐induced mice, and we also collected the liver of LPS‐induced mice to explore the role of miR‐106a‐3p in septic liver dysfunction and its potential molecular mechanisms.

## 2 METHODS AND MATERIALS

### 2.1 Cell culture

The AML12 and LO2 cell lines were bought from Cell Bank of Type Culture Collection, Chinese Academy of Sciences (Shanghai, China). AML12 was cultured in Dulbecco's modified Eagle medium F‐12 (DMEM/F‐12) containing 0.005 mg/ml transferrin (Sigma‐Aldrich), 5 ng/ml selenium (Sigma‐Aldrich), 40 ng/ml dexamethasone (Sigma‐Aldrich), and 90% fetal bovine serum (FBS; Thermo Fisher Scientific, Waltham, MA). The medium also contained 100 IU/ml penicillin and 100 mg/ml streptomycin (Wisent, Nanjing, Jiangsu, China). The LO2 cells were maintained in DMEM containing 90% FBS (Thermo Fisher Scientific), 100 IU/ml penicillin, and 100 mg/ml streptomycin (Wisent); and were incubated at 37°C with 5% CO_2_.

### 2.2 Small interfering RNA and plasmids

The used sequences are listed below. First, miR‐NC, 5′‐ACGGUUAGACCGAUUCCGAAUCCGCG‐3′; miR‐103a‐3p mimic, 5′‐AGCAGCAUUGUACAGGGCUAUGA‐3′; anti‐miR‐103a‐3p (or miR‐103a‐3p inhibitor), 5′‐UCAUAGCGGAUCAAUGCU‐3′. The miR‐NC and anti‐miR‐103a‐3p were constructed into adeno‐associated virus (AAV) by Hanbio (Shanghai, China). Second, si‐NC, 5′‐CTCTGCTCTTAAAGATAATTT‐3′; si‐FBXW7, 5′‐GGGCAGCAGCGGCGGAGGA‐3′; and they were constructed into AAV named AAV‐sh‐NC and AAV‐sh‐FBXW7, respectively. Third, AAV‐FBXW7 contained the full length of FBXW7 mRNA and was used to overexpress FBXW7, and AAV‐vector was used as its negative control (Hanbio).

### 2.3 LPS‐induced cell model, animal model, and collection of human blood

To determine the role of miR‐103a‐3p and FBXW7 in LPS‐induced liver injury, we established LPS‐induced cell model and animal model, and LPS was bought from Sigma‐Aldrich. First, AML12 and LO2 cells were seeded into 12‐well plates, and they were incubated with 50 μg/ml LPS for 24 hr to be used as cell model. Then, they were transfected with miR‐NC and anti‐miR‐103a‐3p using Lipofectamine 3000 (Thermo Fisher Scientific).

On the contrary, we investigated the role miR‐103a‐3p and FBXW7 in LPS‐induced liver injury of mice. Eight‐week‐old C57 BL/6 male mice were used for further research. The mice, from Experimental Animal Centre of Nanjing University (Nanjing, China), were housed in a specific pathogen‐free place at 25–26°C, a ~50% humidity, and light 12 hr/day. All the animals were approved by the Animal Care and Protection Committee of Nanjing University‐Gulou hospital (SYXK 2004‐0013). In our study, they were randomly divided into two groups: sham group and LPS‐induced group. To produce a model with septic liver failure, the maintained mice were injected intraperitoneally with LPS every day for 21 days (6 mg/kg body weight), and 20 mice were used for each group. Meanwhile, AAV was injected into mice via tail vein every 7 days for 21 days (5 × 10^9^ PFU for each mice).

For determination of miR‐103a‐3p levels in human blood, we collected blood samples from 30 patients with sepsis and 30 healthy controls in a hospital, and this project was also approved by Shanghai Dermatology Hospital (No. 2017‐11‐09‐Shanghai DH). Briefly, the blood samples were collected from people by performing venous blood collection and the blood samples were put into a heparin sodium‐contained anticoagulation tube.

The peripheral blood mononuclear cell was isolated from the collected blood by using EasySep™ Human Monocyte Isolation Kit (STEMCELL Technologies Inc., Shanghai, China). Then, the miR‐103a‐3p was determined using quantitative reverse‐transcription polymerase chain reaction (qRT‐PCR) analysis.

### 2.4 Hematoxylin‐eosin (H&E) staining

The harvested mice liver tissues were immediately fixed in 4% paraformaldehyde (Sigma‐Aldrich). The liver specimens were stained with hematoxylin‐eosin staining post paraffin block and section (Beyotime, Haimen, China) following the standard protocols (Guo, Zhang, Wang, Yu, & Wang, 2017). Finally, the stained liver sections were scored in a blind manner to assess the liver injury.

### 2.5 Quantitative reverse‐transcription polymerase chain reaction

We extracted the total RNA using TRIzol reagent (Thermo Fisher Scientific). To obtain complementary DNA, 0.5 μg total RNA was added for reaction using PrimeScript RT Kit (TaKaRa, Dalian, China). Then, to examine the expression of miRNA and mRNA, we used the SYBR kit (Thermo Fisher Scientific). The U6 was used as an internal control of miR‐103a‐3p, and glyceraldehyde‐3‐phosphate dehydrogenase (GAPDH) was considered as an internal control of mRNAs. The used primers were from Sangon (Shanghai, China), and the sequences of primers are listed below. Human Bax, 5′‐GGGTTGTCGCCCTTTTCTAC‐3′ (Forward), 5′‐AGTCGCTTCAGTGACTCGG‐3′ (Reverse); Human Bcl‐2, 5′‐CTTTGAGTTCGGTGGGGTCA‐3′ (Forward), 5′‐GGGCCGTACAGTTCCACAAA‐3′ (Reverse); Human GAPDH, 5′‐CACCCACTCCTCCACCTTTG‐3′ (Forward), 5′‐CCACCACCCTGTTGCTGTAG‐3′ (Reverse). U6, reverse‐transcription PCR primers, 5′‐GTCGTATCCAGTGCAGGGTCCGAGGTATTCGCACTGGATACGACAAATATG‐3′, and 5′‐TGCGGGTGCTCGCTTCGGCAGC‐3′ (Forward), 5′‐GTGCAGGGTCCGAGGT‐3′ (Reverse); miR‐103a‐3p, reverse‐transcription PCR primers, 5′‐GTCGTATCCAGTGCAGGGTCCGAGGTATTCGCACTGGATTCATCG‐3′, 5′‐AGCAGCATTGTACAGGG‐3′ (Forward), 5′‐GTGCAGGGTCCGAGGT‐3′ (Reverse). Human TNF‐α, 5′‐CTGGGGCCTACAGCTTTGAT‐3′ (Forward), 5′‐GGCCTAAGGTCCACTTGTGT‐3′ (Reverse); Human IL‐1β, 5′‐TGAGCTCGCCAGTGAAATGAT‐3′ (Forward), 5′‐CTTGCTGTAGTGGTGGTCGG‐3′ (Reverse); Human IL‐6, 5′‐CCACCGGGAACGAAAGAGAA‐3′ (Forward), 5′‐CAGGCAACACCAGGAGCAG‐3′ (Reverse); Human CAT, 5′‐TTGAAGATGCGGCGAGACTT‐3′ (Forward), 5′‐TGCTTGGGTCGAAGGCTATC‐3′ (Reverse); Human GSH, 5′‐GGAAAGGCGAACTAGTGTTGG‐3′ (Forward), 5′‐GAATGGGGCATAGCTCACCA‐3′ (Reverse); Human SOD1, 5′‐AGCGAGTTATGGCGACGAAG‐3′ (Forward), 5′‐CTTTGGCCCACCGTGTTTTC‐3′ (Reverse). Mouse Bax, 5′‐ATCCAAGACCAGGGTGGCT‐3′ (Forward), 5′‐CCTTCCCCCATTCATCCCAG‐3′ (Reverse); Mouse Bcl‐2, 5′‐ACTGAGTACCTGAACCGGCA‐3′ (Forward), 5′‐AGGTATGCACCCAGAGTGATG‐3′ (Reverse); Mouse TNF‐α, 5′‐GCACAGAAAGCATGATCCGC‐3′ (Forward), 5′‐GTTTGCTACGACGTGGGCT‐3′ (Reverse); Mouse IL‐1β, 5′‐TGCCACCTTTTGACAGTGATG‐3′ (Forward), 5′‐TGATGTGCTGCTGCGAGATT‐3′ (Reverse); Mouse IL‐6, 5′‐TCCTACCCCAATTTCCAATGC‐3′ (Forward), 5′‐ACGCACTAGGTTTGCCGAG‐3′ (Reverse); Mouse CAT, 5′‐GCCAATGGCAATTACCCGTC‐3′ (Forward), 5′‐GAGTGTCCGGGTAGGCAAAA‐3′ (Reverse); Mouse GSH, 5′‐TTTAGCCTTGCGGGGCG‐3′ (Forward), 5′‐CATACGTCACCACTCGCTCG‐3′ (Reverse); Mouse SOD1, 5′‐GGGAAGCATGGCGATGAAAG‐3′ (Forward), 5′‐CTGATGGACGTGGAACCCAT‐3′ (Reverse); Mouse GAPDH, 5′‐CAGGAGAGTGTTTCCTCGTCC‐3′ (Forward), 5′‐GATGGGCTTCCCGTTGATGA‐3′ (Reverse). All primers were purchased from BGI (Shenzhen, Guangdong, China). The relative RNA levels were calculated following the $2^{-\Delta \Delta C_{t}}$ method.

### 2.6 Western blot analysis

The protein lysates were prepared with protease inhibitors cocktail‐contained radioimmunoprecipitation assay lysate (Beyotime). The protein samples were boiled at 98°C for 10 min. Next, 60 μg of proteins was used for analysis on 10% sodium dodecyl sulfate (SDS)‐polyacrylamide gel electrophoresis (SDS‐PAGE; Bio‐Rad, Hercules, CA). The SDS‐PAGE gels were transferred to 0.45 μm polyvinylidene difluoride (PVDF) membranes (Bio‐Rad) for 45 min. Then, the protein‐carried PVDF membranes were blocked with 2% bovine serum albumin, and they were incubated with the indicated primary antibody overnight. Afterward, the membranes were incubated with horseradish peroxidase‐conjugated secondary antibody for 45 min diluted at 1:50,000 (Bioworld Technology, Nanjing, China). Importantly, the primary antibodies including anti‐GAPDH, anti‐FBXW7, anti‐Bax, anti‐Bcl‐2, anti‐TNF‐α, anti‐IL‐1β, anti‐IL‐6, anti‐CAT, anti‐GSH, and anti‐SOD1 were purchased from Sigma‐Aldrich.

### 2.7 Annexin V‐fluoresceine isothiocyanate (FITC)/propidium iodide (PI) staining

To examine apoptosis of AML12 and LO2 cells, they were seeded in 12‐well plates. They were harvested at 100*g* × 5 min post 48 hr transfection with anti‐miR‐103a‐3p followed by 24‐hr LPS incubation (50 μg/ml). Next, they were washed with PBS for three times. Then, they were resuspended with a staining buffer. They were stained with annexin V/FITC for 15 min and PI for another 5 min in the dark, and this detecting kit was bought from Beyotime. In the end, apoptosis percentage of AML12 and LO2 were determined using Bio‐Rad S3e flow cytometry (Hercules, CA). Four wells were prepared for each group and each assay was repeated for three times independently.

### 2.8 Dual‐luciferase reporter assay

In our study, 6 × 10^4^ cells/well were seeded into 24‐well plates. The AML12 or LO2 were cotransfected with pmirGLO‐FBXW7‐3′UTR‐WT (wide‐type) and pmirGLO‐FBXW7‐3′UTR‐mut (mutant) reporter plasmids, and miR‐NC, miR‐103a‐3p mimics, and anti‐miR‐103a‐3p. We measured the luciferase activity of the mentioned pmirGLO‐FBXW7‐3′UTR after 24 hr of cotransfection employing Dual‐Luciferase Assay Kit (Promega, Madison, WI). Here, the plasmids were synthetized by Sangon (Shanghai, China). The mRNA 3′UTR was from National Coalition Building Institute database (NM_033632 [*Homo sapiens*] and NM_001177773 [*Mus musculus*]).

### 2.9 Determination of caspase‐3 activity

The caspase‐3 activity was determined using GreenNuc™ Caspase‐3 Assay Kit (Beyotime) following the manufacturer's instructions. Briefly, 5 × 10^4^ cells/well were seeded into 96‐well plates. Post 48‐hr transfection with anti‐miR‐103a‐3p followed by 24‐hr LPS incubation (50 μg/ml), the kit‐provided substrates and Ac‐DEVD‐CHO were added into each well. After 30 min incubation in a 37°C incubator with 5% CO_2_, we measured the absorbance of each well using a microplate reader (Bio‐Rad), and we used 485 nm as excitation wavelength and 515 nm as emission wavelength. The blank well was considered as zero calibration.

### 2.10 Statistical analysis

The results were indicated as mean±standard deviation. A *p* < .05 was statistically significant. The data in this study were analyzed statistically using GraphPad Prism 6 (GraphPad Software, CA) and SPSS software package (version 19.0; SPSS Inc., NY). Then, statistical significance was tested by the two‐tailed Student's *t* test for two groups comparisons and one‐way analysis of variance test with posthoc analysis contrasts for multigroup comparisons.

## 3 RESULTS

### 3.1 miR‐103a‐3p is upregulated in the serum and liver of LPS‐induced mice and it is increased in the serum of sepsis patients

**Figure 1:**
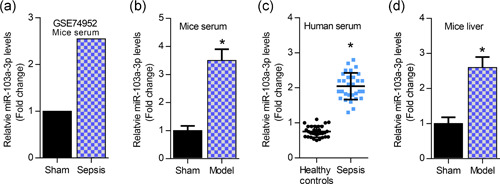
miR‐103a‐3p is elevated in the serum and liver of LPS‐induced mice and it is upregulated in the serum of sepsis patients. (a) GEO2R analysis of miR‐103a‐3p expression in the serum of sepsis mice (GSE74952). (b) qRT‐PCR analysis of miR‐103a‐3p levels in the serum of LPS‐induced mice, *p < .01 versus sham group. (c) qRT‐PCR analysis of miR‐103a‐3p levels in the serum of sepsis patients, *p < .01 versus healthy controls. (d) qRT‐PCR analysis of miR‐103a‐3p levels in the liver of LPS‐induced mice. LPS, lipopolysaccharides; miR, microRNA; qRT‐PCR, quantitative reverse‐transcription polymerase chain reaction. *p < .01 versus sham group

To investigate the role of miR‐103a‐3p in sepsis‐induced liver injury, we determined miR‐103a‐3p expression in the serum and liver of LPS‐induced mice, and we also detected miR‐103a‐3p levels in the serum of sepsis patients. First, GEO2R bioinformatic analysis showed that miR‐103a‐3p was increased in the serum of sepsis mice (Figure 1a). Then, the data showed that miR‐103a‐3p was elevated in the serum of sepsis mice and sepsis patients, evidenced by qRT‐PCR analysis (Figure 1b,c). Similarly, miR‐103a‐3p was upregulated in the septic liver of LPS‐induced mice (Figure 1d).

### 3.2 Knockdown of miR‐103a‐3p inhibits LPS‐induced apoptosis of hepatocytes

**Figure 2:**
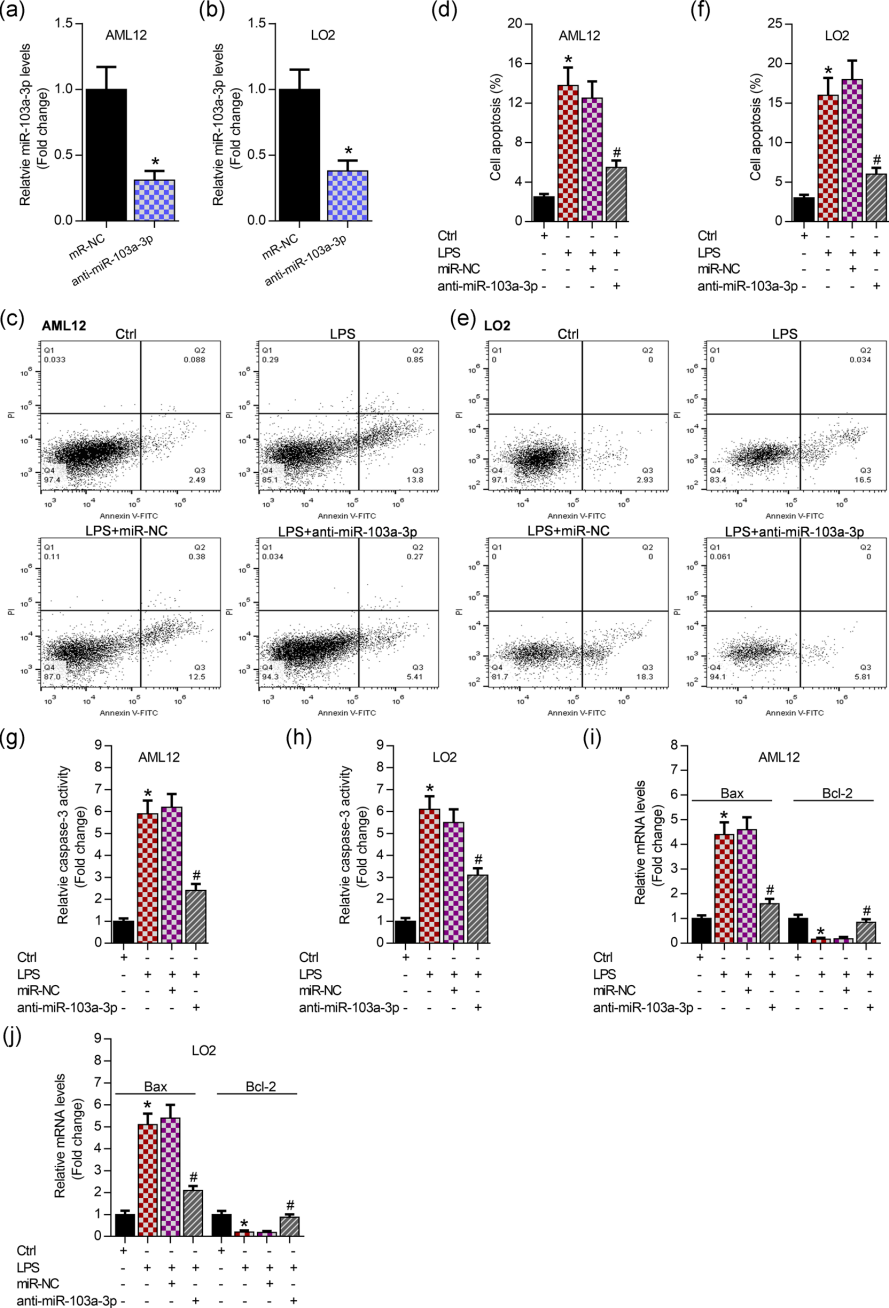
Inhibition of miR‐103a‐3p attenuates LPS‐triggered apoptosis of AML12 and LO2 cells. (a, b) qRT‐PCR analysis of miR‐103a‐3p expression posttransfection with anti‐miR‐103a‐3p in AML12 and LO2 cells, *p < .01 versus miR‐NC. (c, d) Annexin V‐FITC/PI staining showing the apoptosis percentage of AML12 after 48‐hr transfection with anti‐miR‐103a‐3p followed by 24‐hr LPS incubation (50 μg/ml), *p < .01 versus Ctrl group, ^#^ *p* < .01 versus LPS+miR‐NC group. (e, f) Annexin V‐FITC/PI staining showing the apoptosis percentage of LO2 after 48‐hr transfection with anti‐miR‐103a‐3p followed by 24‐hr LPS incubation (50 μg/ml), *p < .01 versus Ctrl group, ^#^ *p* < .01 versus LPS+miR‐NC group. (g, h) Enzyme‐linked immunosorbent assay analysis of caspase‐3 activity in AML12 and LO2 cells after 48‐hr transfection with anti‐miR‐103a‐3p followed by 24‐hr LPS incubation (50 μg/ml), *p < .01 versus Ctrl group, ^#^ *p* < .01 versus LPS+miR‐NC group. (i, j) qRT‐PCR analysis of Bax and Bcl‐2 in AML12 and LO2 cells after 48‐hr transfection with anti‐miR‐103a‐3p followed by 24‐hr LPS incubation (50 μg/ml). FITC, fluoresceine isothiocyanate; LPS, lipopolysaccharides; miR, microRNA, NC, negative control; PI, propidium iodide; qRT‐PCR, quantitative reverse‐transcription polymerase chain reaction. *p < .01 versus Ctrl group ^#^ *p* < .01 versus LPS+miR‐NC group

To explore the effects of miR‐103a‐3p on LPS‐induced apoptosis of hepatocytes in vitro, we knocked down miR‐103a‐3p (Figure 2a,b). It has been reported that LPS always induces apoptosis, inflammation, and oxidation. In this study, the following results demonstrated that LPS obviously increased apoptosis of AML12 and LO2 cells, and interfering miR‐103a‐3p significantly attenuated LPS‐induced cell apoptosis using annexin V‐FITC/PI staining (Figure 2c–f). Then, the knockdown of miR‐103a‐3p markedly decreased caspase‐3 activity evidenced by enzyme‐linked immunosorbent assay in the LPS‐incubated AML12 and LO2 cells (Figure 2g,h). Meanwhile, silencing of miR‐103a‐3p significantly repressed Bax, but enhanced Bcl‐2 expression demonstrated by qRT‐PCR analysis in the LPS‐incubated hepatocytes (Figure 2i,j).

### 3.3 Inhibition of miR‐103a‐3p ameliorates LPS‐induced inflammation and oxidation in hepatocytes

**Figure 3:**
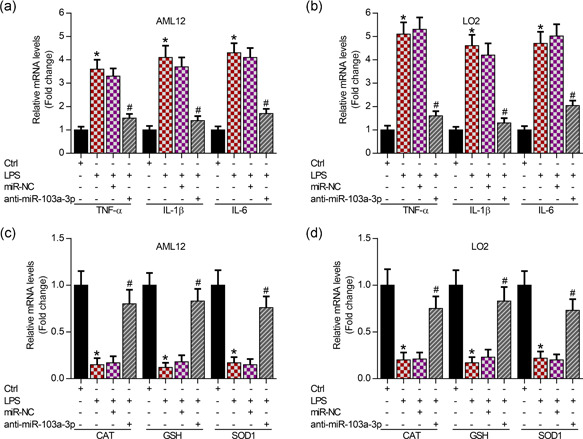
Knockdown of miR‐103a‐3p inhibits inflammation and increases antioxidation activity. (a, b) qRT‐PCR analysis showing the expression of inflammatory factors (TNF‐α, IL‐1β, and IL‐6) in AML12 and LO2 cells post 48‐hr transfection with anti‐miR‐103a‐3p followed by 24‐hr LPS incubation (50 μg/ml), *p < .01 versus Ctrl group, ^#^ *p* < .01 versus LPS+miR‐NC group. (c, d) qRT‐PCR analysis showing expression of antioxidation genes (CAT, GSH, and SOD1) in AML12 and LO2 cells post 48‐hr transfection with anti‐miR‐103a‐3p followed by 24‐hr LPS incubation (50 μg/ml). CAT, catalase; IL, interleukin; GSH, glutathione; LPS, lipopolysaccharides; miR, microRNA, NC, negative control; qRT‐PCR, quantitative reverse‐transcription polymerase chain reaction; SOD1, superoxide dismutase 1; TNF‐α, tumor necrosis factor. *p < .01 versus Ctrl group, ^#^ *p* < .01 versus LPS+miR‐NC group

To determine the role of miR‐103a‐3p in LPS‐induced inflammation and oxidation, we knocked down miR‐103a‐3p in AML12 and LO2 cells. The qRT‐PCR analysis data showed that knockdown of miR‐103a‐3p obviously improved LPS‐mediated inflammatory responses by downregulating mRNA levels of inflammatory cytokines, including TNF‐α, IL‐1β, and IL‐6, in AML12 and LO2 cells (Figure 3a,b). Interestingly, inhibition of miR‐103a‐3p significantly showed antioxidation activity by increasing the expression of antioxidation enzymes, including CAT, GSH, and SOD1, in AML12 and LO2 cells evidenced by qRT‐PCR analysis (Figure 3c,d).

### 3.4 FBXW7 is a downstream target of miR‐103a‐3p

**Figure 4:**
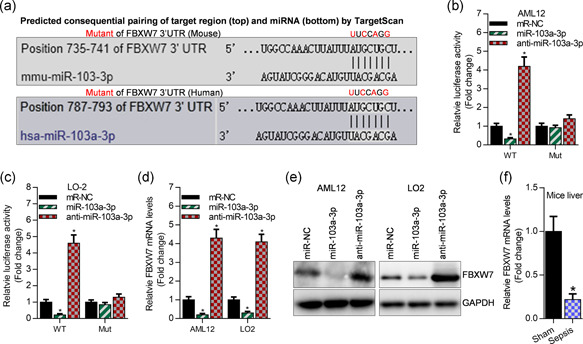
FBXW7 is a downstream target of miR‐103a‐3p. (a) The predicated binding sites of miR‐103a‐3p on FBXW7 3′UTR. (b, c) Luciferase activity of pGLO‐FBXW7 3′UTR post 24 hr of cotransfection with miR‐NC, miR‐103a‐3p, and anti‐miR‐103a‐3p as well as pGLO‐FBXW7‐3′UTR plasmid in AML12 and LO2 cells, *p < .01 versus miR‐NC. (d) qRT‐PCR analysis of FBXW7 expression after 48 hr of transfection with miR‐NC, miR‐103a‐3p, and anti‐miR‐103a‐3p, *p < .01 versus miR‐NC. (e) Western blot analysis of FBXW7 expression after 48 hr of transfection with miR‐NC, miR‐103a‐3p, and anti‐miR‐103a‐3p. (f) qRT‐PCR analysis of FBXW7 levels in the liver of mice. miR, microRNA; NC, negative control; qRT‐PCR, quantitative reverse‐transcription polymerase chain reaction; UTR, untranslated region. *p < .01 versus sham

To detect the potential molecular mechanism of miR‐103a‐3p in sepsis development, we predicated the downstream targets of miR‐103a‐3p using *TargetScan tools*. This bioinformatic analysis showed that FBXW7 is a potential target of miR‐103a‐3p in mouse and human (Figure 4a). Luciferase reporter assay showed that overexpression of miR‐103a‐3p significantly inhibited luciferase activity of FBXW7 3′UTR, and knockdown of miR‐103a‐3p elevated luciferase activity of FBXW7 3′UTR (Figure 4b,c). However, miR‐103a‐3p failed to control the luciferase activity of FBXW7 3′UTR mutant, which contained the mutated binding sites as depicted in Figures 4a–c. Consistently, qRT‐PCR and western blot analyses demonstrated that overexpression of miR‐103a‐3p obviously suppressed the expression of FBXW7 at the mRNA and protein levels, and interfering miR‐103a‐3p increased the expression of FBXW7 (Figure 4d,e). Finally, we observed that FBXW7 mRNA was reduced in the septic liver of LPS‐treated mice (Figure 4f).

### 3.5 Overexpression of FBXW7 improves LPS‐induced liver injury in mice

**Figure 5:**
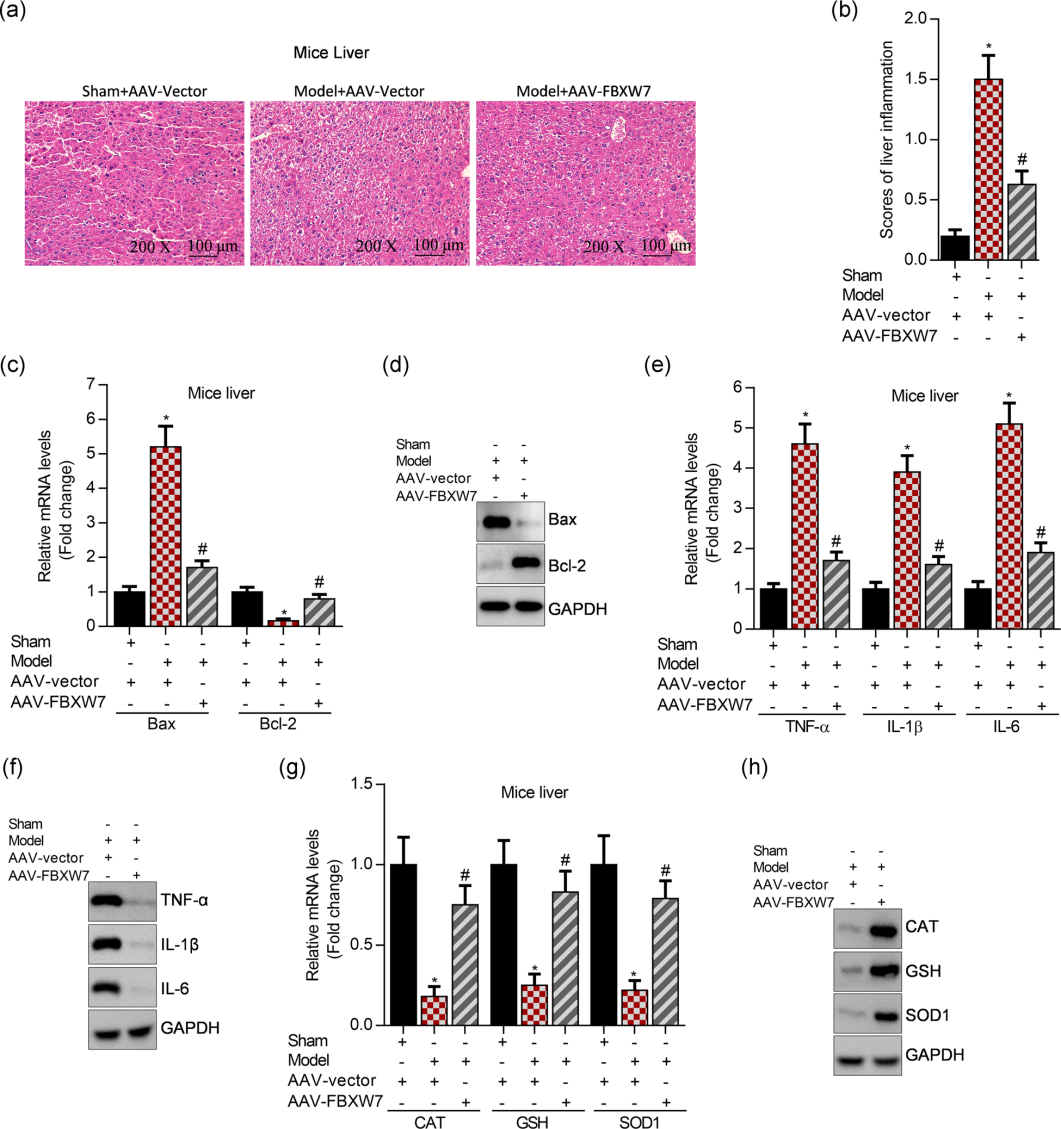
Overexpression of FBXW7 attenuates CLP‐induced liver injury in mice. (a) H&E staining analysis of liver sections at a magnification of 200×. (b) Determination of liver injury according to portal inflammation scores, *p < .01 versus sham+AAV‐vector, ^#^ *p* < .01 versus Model+AAV‐vector. (c) qRT‐PCR analysis of Bax and Bcl‐2 levels in mice liver after 21‐day infection with adreno‐associated virus (AAV), *p < .01 versus sham+AAV‐vector, ^#^ *p* < .01 versus Model+AAV‐vector. (d) Western blot analysis of Bax and Bcl‐2 levels in mice liver after 21‐day infection with AAV. (e) qRT‐PCR analysis of TNF‐α, IL‐1β, and IL‐6 expression in mice liver after 21‐day infection with AAV, *p < .01 versus sham+AAV‐vector, ^#^ *p* < .01 versus Model+AAV‐vector. (f) Western blot analysis of TNF‐α, IL‐1β, and IL‐6 expression in mice liver after 21‐day infection with AAV. (g) qRT‐PCR analysis of CAT, GSH, and SOD1 expression in mice liver after 21‐day infection with AAV, *p < .01 versus sham+AAV‐vector, ^#^ *p* < .01 versus Model+AAV‐vector. (h) Western blot analysis of CAT, GSH, and SOD1 expression in mice liver after 21‐day infection with AAV. AAV, adreno‐associated virus; CAT, catalase; GAPDH, glyceraldehyde‐3‐phosphate dehydrogenase; GSH, glutathione; H&E, hematoxylin‐eosin; IL, interleukin; mRNA, messenger RNA; qRT‐PCR, quantitative reverse‐transcription polymerase chain reaction; SOD1, superoxide dismutase 1; TNF‐α, tumor necrosis factor‐α

To determine the role of FBXW7 in LPS‐induced liver injury, FBXW7 was overexpressed in the liver of mice using AAV. H&E staining showed the morphological alterations of the liver, and LPS significantly enhanced the liver injury (Figure 5a). Overexpression of FBXW7 restored destructive damage of hepatocytes markedly in LPS‐induced septic liver injury of mice (Figure 5a,b). Then, qRT‐PCR and western blot analysis showed that overexpression of FBXW7 significantly suppressed Bax expression and upregulated Bcl‐2 levels, whereas Bax was elevated and Bcl‐2 was suppressed in the septic liver of mice (Figure 5c,d). Furthermore, overexpression of FBXW7 markedly inhibited mRNA and protein expression of inflammatory factors, including TNF‐α, IL‐1β, and IL‐6, in the septic liver of mice (Figure 5e,f). Moreover, overexpression of FBXW7 recovered expression of antioxidation genes, including CAT, GSH, and SOD1, which were significantly decreased in the septic liver of mice (Figure 5g,h).

### 3.6 Knockdown of FBXW7 blocks anti‐miR‐103a‐3p‐improved septic liver injury in LPS‐induced mice

**Figure 6:**
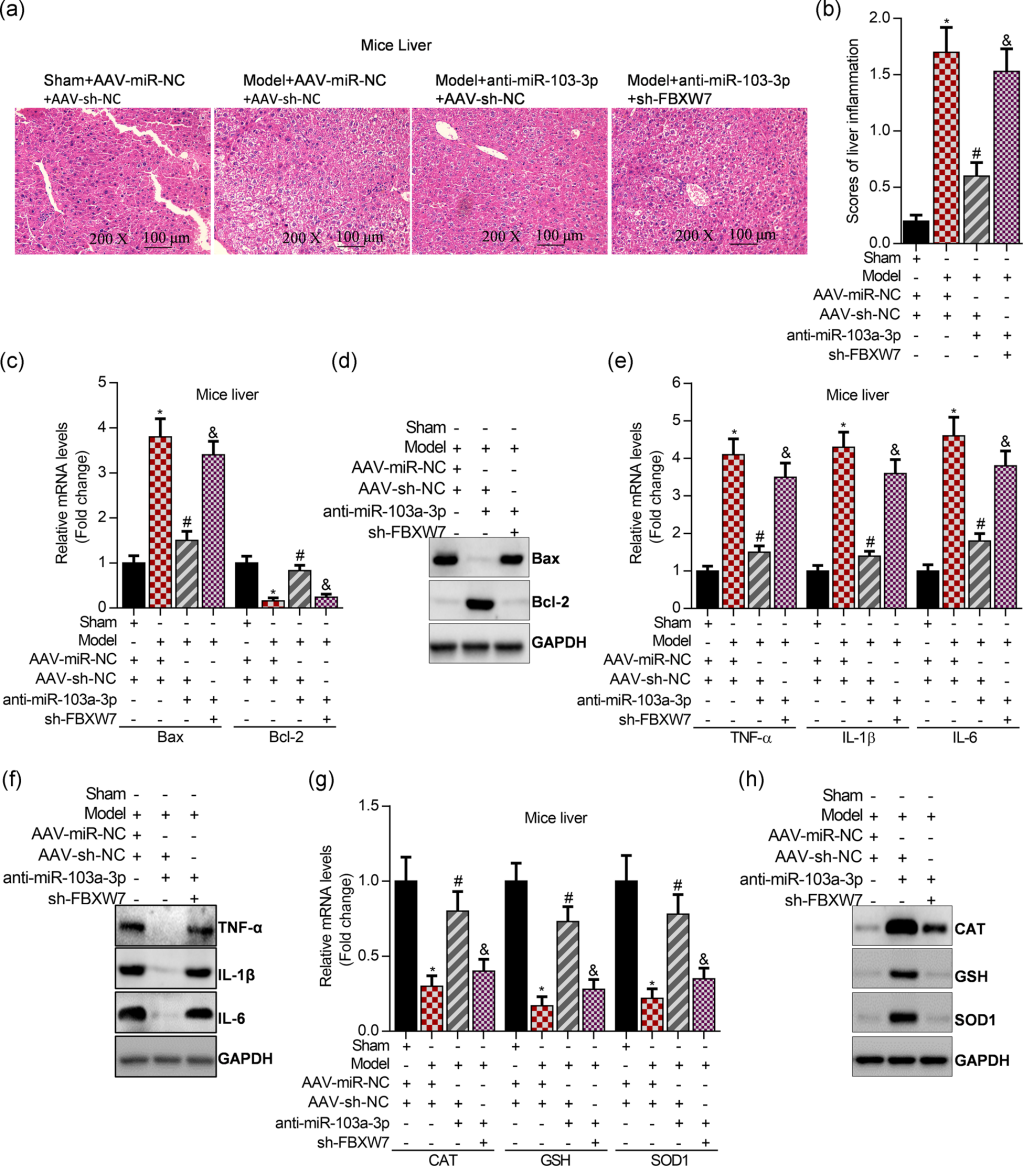
Interfering FBXW7 abolishes anti‐miR‐103a‐3p‐mediated suppression of liver injury in LPS‐induced mice. (a) H&E staining of liver sections at a magnification of ×200. (b) Assessment of liver injury according to portal inflammation scores, *p < .01 versus sham+AAV‐miR‐NC+AAV‐vector, ^#^ *p* < .01 versus Model+AAV‐miR‐NC+AAV‐vector, ^&^ *p* < .01 versus Model+AAV‐anti‐miR‐103a‐3p+AAV‐vector. (c) qRT‐PCR analysis of Bax and Bcl‐2 levels in mice liver after 21‐day infection with AAV, *p < .01 versus sham+AAV‐miR‐NC+AAV‐vector, ^#^ *p* < .01 versus Model+AAV‐miR‐NC+AAV‐vector, ^&^ *p* < .01 versus Model+AAV‐anti‐miR‐103a‐3p+AAV‐vector. (d) qRT‐PCR analysis of Bax and Bcl‐2 levels in mice liver after 21‐day infection with AAV. (e) qRT‐PCR analysis of TNF‐α, IL‐1β, and IL‐6 expression in mice liver after 21‐day infection with AAV, *p < .01 versus sham+AAV‐miR‐NC+AAV‐vector, ^#^ *p* < .01 versus Model+AAV‐miR‐NC+AAV‐vector, ^&^ *p* < .01 versus Model+AAV‐anti‐miR‐103a‐3p+AAV‐vector. (f) Western blot analysis of TNF‐α, IL‐1β, and IL‐6 expression in mice liver after 21‐day infection with AAV. (g) qRT‐PCR analysis of CAT, GSH, and SOD1 expression in mice liver after 21‐day infection with AAV, *p < .01 versus sham+AAV‐miR‐NC+AAV‐vector, ^#^ *p* < .01 versus Model+AAV‐miR‐NC+AAV‐vector, ^&^ *p* < .01 versus Model+AAV‐anti‐miR‐103a‐3p+AAV‐vector. (h) Western blot analysis of CAT, GSH, and SOD1 expression in mice liver after 21‐day infection with AAV. AAV, adreno‐associated virus; CAT, catalase; GAPDH, glyceraldehyde‐3‐phosphate dehydrogenase; GSH, glutathione; H&E, hematoxylin‐eosin; IL, interleukin; mRNA, messenger RNA; qRT‐PCR, quantitative reverse‐transcription polymerase chain reaction; SOD1, superoxide dismutase 1

To determine whether FBXW7 was required for the function of miR‐103a‐3p in septic liver injury, both FBXW7 and miR‐103a‐3p were interfered in the liver of mice using AAV. H&E staining showed the morphological alterations of the liver, and inhibition of miR‐103a‐3p significantly suppressed the septic liver injury, whereas knockdown of FBXW7 obviously abolished anti‐miR‐103a‐3p‐mediated suppression of septic liver injury (Figure 6a,b). Next, interfering miR‐103a‐3p markedly depressed hepatic apoptosis by reducing Bax expression and increasing Bcl‐2 levels in the septic livers, and however, knockdown of FBXW7 obviously blocked anti‐miR‐103a‐3p‐suppressed cell apoptosis in the septic livers of LPS‐induced mice by regulating the expression of Bax and Bcl‐2 (Figure 6c,d). Furthermore, inhibition of miR‐103a‐3p significantly attenuated hepatic inflammation by downregulating expression of TNF‐α, IL‐1β, and IL‐6, and interestingly, this downregulation could be blocked by knockdown of FBXW7 in the septic liver (Figure 6e,f). Finally, inhibition of miR‐103a‐3p significantly ameliorated oxidation by elevating expression of CAT, GSH, and SOD1, and nevertheless, the anti‐miR‐103a‐3p‐mediated antioxidation activity may be reversed by knockdown of FBXW7 in the septic liver (Figure 6g,h).

## 4 DISCUSSION

The molecular mechanism of the LPS‐ or sepsis‐induced acute liver injury has not been completely understood (Jaeschke, 2011). Accumulating studies have reported that LPS induced acute liver injury by promoting apoptosis, inflammation, and oxidation in hepatocytes (Dan et al., 2015; Franco and Cidlowski, 2012; Zhong et al., 2016). The previous reports revealed that several miRNAs have played an important role in sepsis‐induced organ failure, including liver injury (Han et al., 2019; Yuan et al., 2019). For example, miR‐30a inhibits proliferation and promotes apoptosis of hepatocytes via targeting SOCS‐1 in sepsis rats (Yuan et al., 2019) and MCPIP1‐controlled miR‐9 is involved in septic liver injury by regulating SIRT1 expression (Han et al., 2019). In this study, we aimed to explore the role of miR‐103a‐3p in septic liver injury. miR‐103a‐3p always serves as an oncogene (Hong et al., 2014; Hu et al., 2018; Xiong et al., 2017) and it is increased in the plasma of hypertension patients (Bacon et al., 2015; Karolina et al., 2012), and it is able to aggravate angiotensin II‐induced renal inflammation and fibrosis (Lu et al., 2019), implying that miR‐103a‐3p might advance the septic liver failure. Herein, we observed that knockdown of miR‐103a‐3p ameliorated LPS‐induced liver injury by downregulating the expression of apoptotic genes, inflammatory cytokines, and oxidative stress mediators in vivo (in sepsis mice) and in vitro (or in AML12 and LO2 cells). These results indicated a pathogenetic role of miR‐103a‐3p in the liver of septic mice. Therefore, inhibition of miR‐103a‐p may be a promising approach to counter against septic liver injury.

miRNAs may exert their function via targeting the 3′ untranslated region (3′UTR) of mRNA resulting in suppression of target genes. To further uncover the potential molecular mechanisms underlying the pathogenetic role of miR‐103a‐3p in septic liver dysregulation, we focused on the downstream targets of miR‐103a‐3p. Using *TargetScan tools*, miR‐103a‐3p was predicted to specifically target 3′UTR of FBXW7 mRNA. The binding of miR‐103a‐3p with FBXW7 was also confirmed by performing luciferase reporter assay. Meanwhile, we observed that overexpression of miR‐103a‐3p significantly repressed FBXW7 expression and interfering miR‐103a‐3p markedly elevated FBXW7 levels in AML12 and LO2 cells evidenced by qRT‐PCR and western blot analysis. Most interestingly, we found that FBXW7 was decreased in the LPS‐induced septic liver of mice, indicating that FBXW7 might be a protective molecular to improve septic liver failure. The previous studies showed that FBXW7 is an anticancer gene (Fiore et al., 2019; Imura et al., 2014; Li et al., 2019; Xiao et al., 2018; Zhao et al., 2017), and then, FBXW7 suppresses inflammatory signaling via decreasing C/EBPδ and its target gene TLR4 in macrophages and tumor cells (Balamurugan et al., 2013), and FBXW7 also inhibits hepatic inflammation in nonalcoholic fatty liver disease (C. Zhang et al., 2019). In accordance with these previous studies, we observed that overexpression of FBXW7 significantly inhibited cell apoptosis, inflammation, and oxidative stress in the septic liver of LPS‐induced mice. Subsequently, we can conclude that FBXW7 could attenuate the LPS‐induced septic liver failure of mice.

Increasing evidence has pointed out that overactivation of the proinflammatory system appears in septic liver injury, and the proinflammatory cytokines, including TNF‐α, IL‐1β, and IL‐6 are involved in the pathogenesis of septic liver injury (Dan et al., 2015; Jiang et al., 2018; Zhong et al., 2016). Suppressing the production of the proinflammatory factors could ameliorate the severity of septic liver dysfunction (Jiang et al., 2018). For example, inhibition of miR‐155 relieves septic liver injury by inhibiting the JAK/STAT pathway‐mediated expression of inflammatory cytokines (Lv et al., 2015). Sophocarpine attenuates LPS‐induced liver injury by inhibiting inflammation. In this study, downregulation of miR‐103a‐3p significantly had a suppressive effect on the levels of serum TNF‐α, IL‐1β, and IL‐6 in septic mice model, and similarly, it had an inhibitory effect on the expression of TNF‐α, IL‐1β, and IL‐6 in LPS‐incubated AML12 and LO2 cells, which indicated that interfering miR‐103a‐3p might offer a better therapeutic treatment for septic liver failure. However, knockdown of FBXW7 could abolish interfering miR‐103a‐3p‐mediated inhibition of inflammation in the LPS‐induced septic liver injury of mice by regulating the expression of TNF‐α, IL‐1β, and IL‐6. Meanwhile, cell apoptosis is also a contributor to the progression of acute liver injury, and suppression of cell apoptosis could improve liver failure (Yuan et al., 2019). In accordance with our data, silencing of miR‐103a‐3p attenuated LPS‐triggered cell apoptosis in vivo and in vitro by decreasing Bax expression and upregulating Bcl‐2 expression, suggesting that interfering miR‐103a‐3p may improve liver injury by decreasing cell apoptosis. Expectedly, knockdown of FBXW7 could block interfering miR‐103a‐3p‐induced decrease of cell apoptosis. Collectively, we can conclude that miR‐103a‐3p exerts its regulatory activity on inflammation and apoptosis partly relying on FBXW7.

Oxidative stress has been considered as a balance between antioxidant and prooxidant (Sanchez‐Valle, Chavez‐Tapia, Uribe, & Mendez‐Sanchez, 2012). The hydrogen peroxide (H_2_O_2_), superoxide radical ($O_{2}^{•-}$), and nitric oxide (NO) are the chief cause of ROS process in various microenvironments (Funato, Michiue, Asashima, & Miki, 2006; Kiecolt‐Glaser et al., 2003; Slimen et al., 2014). The ROS is always positively associated with septic shock and organ failure, including LPS‐induced liver failure (Zapelini et al., 2008). Therefore, suppression of ROS production could be a promising approach to reverse liver dysfunction. SOD can decrease the levels of ROS and NO (Ren, Yang, Niu, Liu, & Ren, 2011), and CAT can inhibit ROS release via turning H_2_O_2_ into water and oxygen (Azarabadi, Abdollahi, Torabi, Salehi, & Nasiri, 2017), and GSH may protect the liver by repressing the levels of H_2_O_2_. In our study, interfering miR‐103a‐3p significantly reduced ROS content by upregulating expression of SOD1, CAT, and GSH in the LPS‐induced septic liver and in hepatocytes (AML12 and LO2 cells), implying that downregulation of miR‐103a‐3p might be a good choice to attenuate LPS‐induced liver failure by inhibiting ROS production. Nevertheless, we observed that knockdown of FBXW7 obviously reversed the interfering miR‐103a‐3p‐mediated inhibition of ROS production by modulating the expression of SOD1, CAT, and GSH. Consequently, miR‐103a‐3p promotes septic liver injury partly depending on FBXW7.

## 5 CONCLUSION

MiR‐103a‐3p was increased in the serum of sepsis mice and sepsis patients, and it was also elevated in the septic liver of LPS‐induced mice. Further data confirmed that interfering miR‐103a‐3p could decrease LPS‐induced cell apoptosis, inflammation, and ROS both in vitro and in vivo. Moreover, FBXW7 was a direct target of miR‐103a‐3p. Then, FBXW7 was a suppressor of septic liver failure in LPS‐induced mice; overexpression of FBXW7 could significantly attenuate LPS‐induced liver failure in mice. Moreover, knockdown of FBXW7 could markedly block interfering miR‐103a‐3p‐mediated attenuation of LPS‐induced septic liver injury by affecting the expressions of TNF‐α, IL‐1β, IL‐6, SOD1, CAT, GSH, Bax, and Bcl‐2 in mice.

## CONFLICT OF INTERESTS

The authors declare that there are no conflict of interests.

## AUTHOR CONTRIBUTION

Y.‐P. Z. designed the project, offered discussion and suggestions, and provided the financial support. Q. X. performed the animal experiments and she also cultured the cell lines and conducted the cell experiments. Y.‐P. Z. and Q. X. analyzed the experimental data.

## ETHICAL APPROVAL AND INFORMED CONSENT

All applicable international, national, and/or institutional guidelines for the care and use of animals were followed. All procedures performed in our studies involving human participants were in accordance with the ethical standards of the institutional and/or national research committee and with the 1964 Helsinki Declaration and its later amendments or comparable ethical standards. Informed consent was obtained from all included participants.
